# Layers of exposure risk management measures for the prevention and control of infections of healthcare workers treating COVID-19 patients

**DOI:** 10.1371/journal.pone.0332804

**Published:** 2025-09-26

**Authors:** Neştian Ștefan Andrei, Turnea Elena-Sabina, Tiţă Silviu-Mihail, Vodă Ana-Iolanda, Poroch Vladimir

**Affiliations:** 1 Department of Management, Marketing and Business Administration, Alexandru Ioan Cuza University of Iași, Iași, Romania; 2 Grigore T. Popa University of Medicine and Pharmacy, Iași, Romania; Government Villupuram Medical College and Hospital, INDIA

## Abstract

**Background:**

COVID-19 has changed the way hospitals manage infections and how healthcare workers (HCWs) carry out their activities. The pandemic has taught us how safety, hygiene and work schedules should be implemented. After the pandemic, a substantial body of research has examined the managerial capacities of health systems, national prevention programmes, and crisis-response strategies.

**Objective:**

The aim of this study is to determine the most critical risk management measures in the context of COVID-19 pandemic, with a focus both prevention and post-factum control measures.

**Methodology:**

We conducted a study using a questionnaire previously validated by the World Health Organization, adapted to identify the key organisational measures for preventing and controlling infections during interactions with patients infected with COVID-19 virus. The setting for the study was the “Sf. Ioan cel Nou” County Emergency Hospital, Suceava, Romania. A total of 312 responses were collected between 10.10.2020 and 19.03.2021. The study complied with two ethical protocols, data were analysed using 21.0 version of the Statistical Package for the Social Sciences software.

**Results:**

Statistical analysis identified four constructs grouping the measures – technical, organisational, human resources, and others, all aimed at preventing the spread of SARS-CoV-2 within and beyond the hospital. In our vision, these four main resulted constructs could be conceptualised similarly to a security system, which aims protects an “asset” from a threat by applying several layers that gradually reduce risk to near zero. Specifically, the first layer involves managing exposure risk by separating contaminated from uncontaminated areas; the second comprises general preventive measures for healthcare workers operating in contaminated areas; the third layer addresses staff attendance and workplace organisation, and the fourth focuses on risks perceived and manifested at the individual level.

**Conclusion:**

Exposure risk assessment and management follow a logic similar to that of a security system designed to protect an “asset” from a threat. In our case, the health of the HCWs was at risk due to their interactions with COVID-19 patients at “Sf. Ioan cel Nou” County Emergency Hospital, Suceava, Romania. Hypothesis testing indicated that all implemented measures contributed significantly to pandemic management, although a functional exposure risk management system requires effective risk reduction across all 4 layers. Given the validity of the second hypothesis – that preventive measures were generally applied at the hospital – we can conclude that HCWs and managers may primarily focus on this type of preventive measure, mainly addressing the second layer alone. This imbalance risks diverting managerial attention and resources toward the second layer at the expense of the other three.

## 1. Introduction

The COVID-19 global pandemic must be examined from multiple perspectives, reflecting challenges at both microeconomic levels (individuals, families, communities, hospitals, etc.) and macroeconomic levels (local or national public health policies, governments, country population, regional population, public and private companies, World Health Organization, the global community). Key management issues include: i) the capacity of national governments to implement the appropriate legislative measures throughout the pandemic phases (initial pandemic outbreak, periods of high or low case numbers, population vaccination, the COVID-19 pandemic conclusion), communicate effectively with a concerned public, allocate necessary governmental resources for each stage, and assess the outcomes of such system-wide measures; ii) the managerial and procedural capabilities of hospitals (Healthcare Facilities Management – HFM) to make and execute probabilistic decisions amid uncertainty, including unclear disease symptoms, evolving treatment protocols, shortages of protective equipment, surging patient loads and limited material and human resources, and intense health system pressures; iii) the engagement of medical personnel – ranging from health ministers and national and local public health authorities to hospital management, doctors, nurses, caregivers – in identifying COVID-19’s causes and symptoms to guide interventions minimising the number of affected individuals; iv) public willingness to trust and comply with restrictive measures taken by authorities despite limitations on individual freedoms; v) other contributing factors.

Nearly three years after the end of the COVID-19 pandemic, there remains a pressing need for research in social and medical sciences examining the managerial response capacities of systems – including governments, public entities, or hospitals – in responding to crises with an impact on the entire population, such as pandemics, wars, or natural disasters. This includes evaluating national prevention and response strategies for health emergencies, natural catastrophes (earthquakes, floods, hurricanes), or regional or global conflicts; developing diagnostic methods to assess healthcare facilities’ operational readiness; and analysing decision-making processes that enhance responsiveness through maintaining adequate reserves of medical supplies and human resources. Furthermore, research should address the formulation of clear procedures via local or national communication campaigns, as well as the psychological burden on the population required to make rapid decisions and adapt behaviour during global crises.

After analysing the Healthcare Facilities Management (HFM) during the pandemic, the study proposes a layered security system approach [1–4] comprising four clusters: the separation of COVID-19 and non-COVID patient flows; implementation of protective measures for personnel – including doormen, receptionists, caregivers, nurses, and doctors – when interacting with COVID-19 patients; operational management aimed at minimising infection rates among hospital staff; workforce motivation and engagement strategies addressing psychological, social, and financial factors, alongside individual health surveillance such as testing, quarantine, activity reduction.

The concept of a *layer* originated from the need to analyse interactions among elements within complex structures or graphical models, facilitating the extraction of subsystems of interest [5] for an in-depth study of the components. Widely applied across disciplines such as computer networks, fluid mechanics, and aerodynamics – where distinct differences appear between layers – this concept has also been adopted in management. In the context or prevention measures, identifying deep layers is crucial, as differences exist in measurement criteria: employees often focus on patient numbers, whereas management prioritises the attitudes of government bodies and their financing of this difficult process [6,7]. A multi-layered approach adds complexity to logical framework model but is essential for improving managerial efficiency [8]. Using a framework for managing complex business systems involving multiple actors requires the identification and thorough analysis of interlinked process layers [9] to enable managerial interventions during the COVID-19 pandemic.

The paper is organized into five main sections to provide a logical and coherent presentation of the research process and findings. Section 1 and 2 offer a brief overview of the key risk management measures employed during the COVID-19 pandemic. Section 3 (Methodology) outlines the research design, data collection methods, and analytical approaches used. Section 4 presents the main results, while section 5 discusses the findings in relation to the study objectives and existing literature, and draws the main conclusions.

## 2. Literature review

### 2.1 Management of the division (contaminated and uncontaminated, COVID/ NON-COVID) areas

The global rise in COVID-19 cases necessitated urgent and substantial reorganization of healthcare services. Acute care hospital facilities were rapidly repurposed to accommodate patients testing positive for SARS-CoV-2. As highlighted by Scichilone et al. (2020) [10] and Hugelius et al. (2021) [11], this paradigm shift required rapid, large-scale decisions. Hospitals implemented strict protocols to separate contaminated (COVID) and uncontaminated (non-COVID) areas. These included designated circulation spaces for COVID-19 patients (CIRCUL_SPACE) and clearly defined treatment zones (DELIMIT_SPACE). Visual aids such as signage, maps, and diagrams were used to guide patient movement (SIGN_MAP), alongside efforts to maintain a continuous supply of sanitary materials (SUPPLY_MADE). Additionally, hospitals facilities established three distinct zones: contaminated, potentially contaminated, and uncontaminated) (AREAS3). Also, specialised training was provided to medical staff (TRAINING_EMPLOYEE). Access within hospitals was restricted to patients and authorised personnel only (FORBID_ENTER), and staff schedules were reorganised to minimize the risk of cross-contamination (SHIFT_STAFF).

This section examines eight organisational strategies hospitals adopted during the pandemic: circulation management (CIRCUL_SPACE); spatial separation of COVID vs non-COVID zones (DELIMIT_SPACE); visual signs, maps, or diagrams to guide patients, visitors, and staff (SIGN_MAP); consistent sanitary supply chains (SUPPLY_MADE); structured contamination risk zones – contaminated, buffer, and uncontaminated (AREAS3); COVID-19 specific staff straining (TRAINING_EMPLOYEE); access control measures (FORBID_ENTER); and adjusted shift scheduling (SHIFT_STAFF).

One key strategy involved relocating the existing inpatients to “COVID-free “ areas and assigning specific facilities *–* or entire hospitals *–* for COVID-positive patients. Hospitals established three main areas: 1. *Contaminated zones for confirmed COVID-19 patients*; 2. *Buffer or grey zones –* for patients potentially contaminated; and 3. *Uncontaminated zone* for patients testing negative. These buffer zones played a crucial role in managing suspected respiratory cases, while awaiting test results, and required proper ventilation and fully equipped staff with masks, gloves and face shields. However, in regions experiencing surges in hospital admissions, maintaining these conditions proved difficult [10]. Overcrowding hindered adherence to physical distancing and ventilation protocols [12,13]. Additionally, the rapid increase in demand for testing and treatment strained human and material resources, including personal protective equipment (PPE) and qualified medical staff. Beyond the physical risks associated with the care provided for patients with highly contagious diseases, healthcare professionals also faced substantial psychological stress from working in overburdened systems while caring for highly infectious patients [14].

During the COVID-19 pandemic, numerous strategies were implemented to minimize transmission risks, including restricted visits, telemedicine and telecommuting where possible. Hugelius et al. (2021) [11] reported that only patients and authorized medical personnel were allowed access to medical facilities, helping to reduce crowding [11]. Routine visits were rescheduled, and non-essential procedures were delayed (or postponed) during the pandemic’s peak. These measures not only decreased congestion in workplace and clinical areas but also helped patient flow management. To further support infection control and prevention, signage systems were introduced throughout hospitals [15]. This included: signs, maps, and diagrams designed to enhance compliance with infection control protocols by guiding patients, visitors, and staff through designated routes [15]. Such visual aids were strategically placed to facilitate navigation and minimize unnecessary exposure [16].

Despite centralized strategies, local adaptations varied significantly. Hospital management or local authorities often adjusted protocols to fit regional needs, reallocating wards, staff or equipment as necessary [17]. Many institutions adopted structured models based on strict protocols that enabled the simultaneous identification and treatment of both COVID-19 and non-COVID-19 patients [10]. Interestingly, other studies have suggested that the presence of COVID-19 did not significantly affect the location of hospital-acquired pressure injuries (PIs) [18]. These findings underline that factors such as immobility, prolonged bed rest, and reduced tissue perfusion may play a more prominent role than the presence of COVID-19 itself in determining injury location [18].

The *reorganisation of medical staff scheduling* was another strategy used to reduce the risk of healthcare-associated infections [19]. Shifts were carefully structured to minimise the potential for cross-contamination between colleagues working different shifts. This proactive measure aimed to strengthen infection control protocols within the hospital, protecting both staff and patients from viral transmission [19].

Many healthcare facilities faced shortages of vital supplies during the pandemic, including alcohol-based hand rub (ABHR) and personal protective equipment (PPE), particularly masks, which were essential equipment for infection prevention and control (IPC). Major disruptions in global shipping, combined with a sudden surge in demand, exacerbated these shortages, leaving many facilities ill-equipped. To address these challenges, many countries collaborated with local industries, providing capital investment, assistance with raw material procurement and distribution, and legal support to streamline production. Regulatory frameworks were temporarily modified to facilitate efficient production and distribution of medical supplies during the COVID-crisis [20]. Additionally, hospitals implemented specialised COVID-19 training for staff; however, the quality of training varied. In some cases, medical personnel lacked sufficient knowledge to care for critically ill COVID-19 patients [21], highlighting a gap between preparedness and practical competence.

Based on the literature, we formulated the following hypothesis:

*Hypothesis 1. Division of hospital areas into contaminated and uncontaminated zones, as well as into COVID and non-COVID sections, played an important role in managing the pandemic.*

### 2.2. General preventive measures for HCWs working in contaminated areas

In this study, we considered four main items designed to maximise protection for healthcare workers (HCWs) operating in high-risk, contaminated areas [22]. These include [22]:

1. The consistent and rational use of personal protective equipment (PPE) when HCWs are exposed to COVID-19 patients (RATIONAL_PPE);
2. Strict adherence to the “*five important moments for hand hygiene*” – before patient contact, before aseptic procedures, after exposure to body fluids, and after contact with patient surroundings, and after patient contact (RULE_5HH);
3. Enhanced protective measures against airborne transmission during aerosol-generating procedures for patients suspected or confirmed to have COVID-19 (PROTECT_AGP);
4. Enforcement of respiratory prevention measures, including wearing masks, using disposable tissues when sneezing, and washing hands after contact with nasal or oral secretions (RESPIRATORY_PREV).

During the pandemic, significant efforts were made to improve the use of personal protective equipment (PPE) and compliance with safety guidelines during interactions with COVID-19-infected patients [23]. These efforts included enhancing medical procedures and protocols, developing training modules for healthcare workers, and implementing enforcement mechanisms to ensure strict adherence to PPE and safety guidelines. Regular evaluations, active feedback, educational programs, and mass campaigns were designed and implemented to minimize infection risk and protect both patients and healthcare professionals. In response to the ongoing COVID-19 pandemic, rigorous protective protocols were introduced to mitigate airborne transmission during aerosol-generating processes for suspected or confirmed COVID-19 cases. According to Gomes et al. (2022) [24] and Obaid et al. (2023) [25], these measures formed part of a comprehensive strategy to promote compliance with infection prevention and control policies. To further reduce virus transmission, healthcare facilities also improved their infrastructure and support systems, including equipment, accessibility, information technologies, and sustainability measures [26,27]. An effective infection prevention and control (IPC) strategy is mandatory for maintaining essential healthcare services and protecting medical personnel and patients. Non-compliance with IPC measures can lead to increased morbidity and mortality rates, prolonged hospitalisations, and higher healthcare costs [24].

Respiratory hygiene was also a key component of SARS-CoV2 infection control transmission. Measures included wearing masks – such as NIOSH-approved, fit-tested N95 filtering facepieces – using disposable tissues when sneezing or coughing, and performing routine handwashing after contact with respiratory secretions [28]. Eye protection, such as shields or goggles, was recommended during all clinical encounters in areas with substantially high transmission rates, as well as the use of any equipment serving as a barrier against direct mucous membrane exposure and hand-to-eye contamination [29]. Such precautions were essential for preventing the spread of respiratory illnesses within healthcare facilities.

The “*Five Moments for Hand Hygiene*” remained a cornerstone of preventive practice, helping to minimize infection risk in healthcare settings [30,31]. Adherence to these practices protected both healthcare providers and patients from potential infections. Healthcare professionals in clinical settings have been closely monitored for compliance with five hand-hygiene moments, and such monitoring has gained traction as a means of prioritising safer environments for both staff and patients [32].

Based on the literature review, we formulated the following hypothesis:

*Hypothesis 2. General preventive measures applied in direct patient care were* *the most frequently implemented in the analysed hospital.*

### 2.3. General staff management measures for HCWs working in contaminated areas

During the COVID-19 pandemic, hospitals implemented several supportive measures to assist healthcare staff engaged in frontline care [33]. These measures aimed to reduce occupational risk, maintain staff morale, and ensure continuity of care in high-pressure environments. Key actions included:

1. Provision of designated living spaces (LIVING-SPACE) for those in direct contact with COVID-19 patients. On-site accommodation not only reduced the risk of transmitting the virus to family members but also provided a much-needed rest area close to the workplace, helping personnel reduce fatigue [34].
2. Psychosocial assistance (PSYCH_ASSIST) for staff during quarantine or illness. Support included such psychological counselling provided on-site or via online sessions to help personnel cope with stress, anxiety, and emotional exhaustion resulting from the high demands needed of COVID-19 specialized care. Some hospitals created peer support groups where personnel could discuss their problems, learn how to deal with anxiety, or build resilience and reduce feelings of isolation [35–37].
3. Financial compensation (FINANCIAL_COMPENS) for quarantine periods and sick leave. Many healthcare facilities ensured full pay during periods of quarantine or illness, reducing financial stress and allowing staff to stay focused on patient recovery and prevention measures without concerns about income loss [38].
4. Advanced training programs (TRAINING_PREV) on infection prevention and control. To decrease the spread of the virus, medical establishments implemented advanced training and education programs to strengthen staff capacity in handling infection prevention and control [39,40]. Some examples may include simulation drills and other practical exercises; digital learning modules focused on infection control, and advanced Intensive Care Units (ICU) which provided to staff the most recent information on best practices for COVID-19 prevention [41].
5. Compulsory or voluntary leave for non-COVID staff (CV_VACATION) to prevent burnout and ensure workforce redistribution [39,40]. To reduce staff, burden and manage hospital resources effectively, medical professionals not involved directly in COVID-19-related activities were placed on either compulsory or voluntary leave. This precautionary measure prevented staff fatigue, gave flexibility in workforce management and enabled redeployment of personnel, no rest being needed.

The COVID 19 crisis posed extreme physical and psychological challenges for healthcare workers (HCWs). Responsibilities included isolating patients, implementing and monitoring infection prevention and control measures, and adhering to strict protocols, while managing emotional strain, fear of getting infected, and high stress [42–45]. These pressures increased the risk of adverse mental and physical health outcomes, including depression, anxiety, and sleep disturbance [45].

Recognition and reward were also critical. Support for healthcare workers took *extrinsic forms*, such as financial incentives, pay increases, and bonuses, and *intrinsic forms,* including satisfaction from patient recovery, positive feedback, and better self-esteem [46,47]. However, several studies highlighted persistent challenges, including burnout, shortages of personal protective equipment, inconsistent communication, and reduced rewards [48]. According to Brolan et al. (2022) inequities in pay were a significant concern among local HCWs [49]. In some cases, employees hired by international agencies or donors received higher salaries than locally contracted counterparts, leading to frustration, demoralization, and workplace tension. Dissatisfaction also arose when promised government bonuses or supplementary payments were delayed or not delivered. Similarly, Gavric et al. (2023) reported that many HCWs felt undervalued, believing they were not adequately compensated for their work [50].

Drawing on above findings from the literature, we formulated the following hypothesis:

*Hypothesis 3. Implementation of comprehensive support measures — including designated living spaces, psychological assistance, financial compensation, advanced training, and flexible leave options — were important in managing the pandemic.*

### 2.4. Managing the health condition of HCWs working in contaminated areas

In response to the COVID-19 pandemic, healthcare institutions implemented a range of proactive measures to safeguard healthcare workers and minimise the risk of virus transmission.

These included:

a) **Suspension of patient interaction** (SUSPENSION_INTERACTION). A first step in managing health of healthcare workers (HCWs) working in contaminated areas included *suspension of any interaction* with patient care for 14 days after unprotected exposure to a confirmed COVID-19 patient. This precaution aimed to prevent potential transmission from asymptomatic infected staff to patients and staff, thereby protecting both the healthcare environment and the community at large [51].
b) **Mandatory quarantine** (QUARANTINE). Exposed healthcare workers were required to undergo a *14-day quarantine* to allow for symptom development and prevent possible transmission to colleagues and patients. This measure was important to prevent virus spread and not allow enough time for the virus to manifest itself. During this period, they were monitored for clinical signs of infection [52].
c) **COVID-19 testing and self-monitoring** (TEST_COVID19; SELFM_TEMP and INSTRUCT_SYMPT). *Regular COVID-19 testing* was conducted to detect asymptomatic infections early and enable timely isolation, protecting not only the medical staff but also the patients present in the healthcare facility [53]. Staff were also instructed to monitor their temperature and respiratory symptoms daily for 14 days post-exposure, (SELFM_TEMP), and seek medical assistance immediately if symptoms developed (INSTRUCT_SYMPT) [54]. The urgent need to report and early intervention may lead to better outcomes for the individuals and the team, the virus not being spread further within the healthcare environment.
d) ***Enhanced contact and exposure precautions*** (PREVENT_CONTACT_EXPOSURE). The implementation of enhanced preventive measures to reduce contact and splash exposure during the care of patients with acute respiratory diseases, alongside standard precautions applied to all patients [55,56]. Enhanced preventive measures must be followed to protect HCWs from potential exposure to infectious agents. These include correct use of PPE, adherence to strict hygiene practices, and maintaining physical distancing when appropriate. Minimizing exposure risk supports a safer working environment, which is crucial during high-risk events such as a pandemic.

Based on the previous literature review, we formulated the following hypothesis:

*Hypothesis 4. The implementation of comprehensive health management measures for healthcare workers (HCWs) in contaminated areas, including suspension of patient care interactions after unprotected exposure, mandatory quarantine, regular COVID-19 testing and self-monitoring, and enhanced preventive practices, was crucial for managing the pandemic.*

## 3. Methodology

This research is based on a previously developed questionnaire for exposure risk assessment and management developed by the World Health Organization, applied to health care workers (HCWs) [39]. The instrument provided general guidance and was used by hospitals worldwide for internal purposes: (1) to categorise the risk level of each healthcare worker after exposure to a COVID-19 patient, and (2) to guide the management of exposed HCWs [39]. We adapted the questions in the questionnaire to identify the main organisational measures that prevented and controlled infections during interactions with COVID-19 patients at “Sf. Ioan cel Nou” County Emergency Hospital in Suceava in Romania. This hospital was selected because it experienced the first major COVID-19 outbreak in Romania early in the pandemic.

Data were collected during 10 October 2020 and 19 March 2021, yielding 312 responses from medical staff, primarily doctors, residents and nurses. Two ethical protocols were approved for this study: one by three parties – National Authority for Quality Management in Health, Grigore T. Popa University of Medicine and Pharmacy, Iași, and Alexandru Ioan Cuza University of Iași (Approval number 12677, dated 27 July 2020), and the other one given prior to data collection data from “St. Ioan cel Nou” County Emergency Hospital with an approval from Grigore T. Popa University of Medicine and Pharmacy, Iași, Romania (approved 15 June 2020). All respondents had contact with patients diagnosed with COVID-19. The hospital management distributed the on-line questionnaire to employees. At the start of the survey, participants were informed that participation was voluntary.

Data were analysed using SPSS software version 21.0. Exploratory factor analysis using principal component analysis was conducted alongside descriptive statistical analysis. The first method facilitated grouping the responses into constructs, simplifying interpretation and drawing conclusions.

## 4. Results

Descriptive statistics and principal component analysis were used in this study.

All the questions are presented in Table 1. And were later grouped into four main constructs to illustrate the organizational measures that collectively helped the hospital manage the pandemic. The questions referred to the three months preceding questionnaire completion. Responses were recorded on a scale 1 (minimum) to 5 (maximum) to assess the intensity of organizational measures implemented during the pandemic.

**Table 1:** Descriptive statistics for the questions used in the research (N = 312).

No. crt.	To what extent do you believe the following stipulations have been implemented and adhered to in the hospital where you work during the past 3 months?	Factor code	Mean	Std. Deviation
1	Staff have been instructed to contact a medical service if any COVID-19 symptoms occuryes	INSTRUCT_SYMPT	4,91	0,382
2	Respiratory prevention measures have been applied (wearing a mask, using disposable wipes for sneezing, washing hands after contact with nasal or oral secretions, etc.).	RESPIRATORY_PREV	4,91	0,43
3	Three areas have been delimited: COVID patient area (contaminated), a potentially contaminated area (buffer) and an uncontaminated area	AREAS3	4,91	0,418
4	The rule of the “5 important moments for hand hygiene” was applied before contacting the patient, before performing aseptic procedures, after exposure to body fluids, after touching the patient or after being in contact with objects near the patient	RULE_5HH	4,88	0,476
5	The circulation spaces for patients with COVID-19 and their dedicated care staff were delimited by the circulation spaces dedicated to other patients	CIRCUL_SPACE	4,88	0,416
6	It was forbidden to any person other than patients and medical staff to enter the sanitary unit	FORBID_ENTER	4,87	0,519
7	The rules for rational, correct, and regular use of personal protective equipment in the exposure to patients with COVID-19 have been strengthened	RATIONAL_PPE	4,86	0,476
8	The spaces for diagnosing and treating patients with COVID-19 were delimited from the spaces for treating other patients	DELIMIT_SPACE	4,86	0,494
9	Supplies were constantly made for sanitary materials/ objects, etc. useful in managing COVID-19 cases	SUPPLY_MADE	4,85	0,494
10	Protective measures have been strengthened in the case of air transmission for aerosol generating procedures for all patients suspected or confirmed with COVID-19	PROTECT_AGP	4,83	0,492
11	Preventive measures that reduce contact and splash exposure during the care of all patients with acute respiratory disease have been strengthened, as well as the standard precautions during the care of all patients	PREVENT_CONTACT_EXPOSURE	4,82	0,532
12	Signs/ maps/ diagrams were displayed to follow the routes through each type of area	SIGN_MAP	4,79	0,612
13	Daily self-monitoring of temperature and respiratory symptoms for 14 days after unprotected exposure to a patient with COVID-19	SELFM_TEMP	4,75	0,774
14	Specialised trainings regarding COVID-19 were conducted for the hospital employees	TRAINING_EMPLOYEE	4,7	0,81
15	The shifts of the medical staff were reorganised to reduce the risk of contamination from colleagues in other shifts	SHIFT_STAFF	4,51	1,067
16	Quarantine for 14 days	QUARANTINE	4,44	1,185
17	Suspension of any interaction regarding patient care for a period of 14 days after unprotected exposure to a confirmed COVID-19 patient	SUSPENTION_INTERACTION	4,41	1,205
18	Testing for COVID-19 viral infection	TEST_COVID19	4,38	1,14
19	Advanced training/ education programs for the prevention and control of infections for health care staff	TRAINING_PREV	4,28	1,122
20	Special living spaces have been arranged within the hospital for the medical staff that had direct contact with COVID-19 patients	LIVING_SPACE	3,62	1,674
21	Providing psychosocial assistance to staff in the unit during their quarantine or during the disease, if a confirmed case of COVID-19 occurs	PSYCH_ASSIST	3,55	1,531
22	Providing financial compensation/ indemnities for the quarantine period and for the duration of the disease	FINANCIAL_COMPENS	3,01	1,724
23	A part of the medical staff that does not directly deal with activities related to COVID −19 has been sent on compulsory or voluntary vacation	CV_VACATION	2,55	1,666

*Note: The individuals responded to each question with scores from 5 (Always) to 1 (Never). These terms were explained, as it follows: **Always** = more than 95% of the time; **Most of the time** = from 50% to 95% of the time; **Occasionally** = from 20% to less than 50% of the time; **Rarely** = less than 20% of the time; **Never**. Source: authors’ contribution.

All questions recorded mean scores above the midpoint of the 1–5 scale. Most items (19 out of 23) had mean scores exceeding 4 out of 5, indicating that the measures were implemented between 50% and 95% of the time. The highest score (4,91 out of 5) was observed for three items: HCWs were trained to contact a medical service if COVID-19 symptoms developed; respiratory prevention measures were applied (such as wearing a mask, using disposable tissues when sneezing, and handwashing after contact with nasal or oral secretions); and the hospital space was divided to three areas (contaminated area, buffer zone, and uncontaminated area). These measures were likely the most widely adopted due to their cost-effectiveness, ease of implementation, and proven efficacy in practice during the pandemic.

Two additional items had mean scores of 4,88 out of 5, showing that the medical staff at “Sf. Ioan cel Nou” County Emergency Hospital in Suceava consistently used the 5 most important moments for hygiene and prevention, and maintained separation of COVID and NON-COVID circulation areas. With a mean of 4,87 out of 5, hospital access was strictly restricted to medical staff and patients. This score was somewhat lower than expected, given that such restrictions were applied across all COVID-19 hospitals in Romania.

The strengthening of the rules for rational, correct, and consistent use of PPE during exposure to COVID-19 patients generated a mean of 4,86 out of 5, which effective management in providing the supplies and their use, despite the increase costs of disinfectants, disposable masks and gloves, and other cleaning and hygiene products for hospitals during the pandemic. Special attention was given to protective measures during AGPs applied to (possible) COVID-19 patients due to the risk of airborne transmission. Correspondingly, the item addressing protection in this context scored highly (PROTECT_AGP: Mean = 4,83 out of 5, Std. dev. = 0,492).

A higher mean was anticipated for the item Advanced training/ education programs for the prevention and control of infections for healthcare staff (TRAINING_PREV Mean = 4,28 out of 5; Std. dev. = 1,122). However, the relatively large standard deviation of 1,122 indicates considerable variation in responses, participants rating the item with possible maximum scores or lower by 1,122 than the mean. So, we have rather non-homogeneous answers, which resulted in a mean above 4 out of 5.

The lowest results were for: compulsory or voluntary vacation of the medical staff that does not directly deal with activities related to COVID −19 (CV_VACATION: Mean = 2,55 out of 5; Std. dev = 1,666), provision of financial compensation/ indemnities for the quarantine period and for the duration of the disease (FINANCIAL_COMPENS: Mean = 3,01 out of 5; Std. dev = 1,724), provision of psychosocial assistance to staff in the unit during their quarantine or during the disease, if a confirmed case of COVID-19 occurred (PSYCH_ASSIST: Mean = 3,55 out of 5; Std. dev. = 1,531), and for special living spaces arranged within the hospital, for the medical staff that had direct contact with COVID-19 patients (LIVING_SPACE: Mean = 3,62 out of 5; Std. dev. = 1,674), with the observation that these items had the highest values from Std. dev. from the whole items.

**Exploratory factor analysis using principal component analysis**

Table 2 explains the total variance of the 23 variables used in this study.

**Table 2:** Total variance explained for the four constructs.

Component	Total	% of Variance	Cumulative %
1	7,419	32,255	32,255
2	2,582	11,227	43,482
3	1,934	8,408	51,890
4	1,406	6,111	58,000

Note: Extraction method: Principal component analysis. Source: authors’ contribution.

Component extraction was repeated until the factor loadings adequately explained the constructs. This process yielded four useful components for our analysis, grouping technical, organizational, human resource, and other measures aimed at preventing SARS-CoV-2 transmission within and beyond the hospital. The total variance is explained by the four constructs, as follows: the first construct explains 32,255% of the variance, the second 11,227% of the variance, the third 8,408% of the variance, the last explaining 6,111% of the variance. Together, the four constructs explain 58% of the total variance, with the observation that the first construct justifies the highest proportion.

The following figure presents the four constructs with their individual Eigenvalues.

Table 3 illustrates the main indicators which validate the factor analysis

**Table 3:** KMO and Bartlett’s test.

Kaiser-Meyer-Olkin Measure of Sampling Adequacy		0,864
Bartlett’s Test of Sphericity	Approx. Chi-Square	3549,730
	df	253
	p value	0,000

Source: authors’ contribution.

The Kaiser-Meyer-Olkin value was (0,864 > 0,5), the Bartlett test (3549,730), and the p value p = 0,000 (< 0,01), conveying that there is a strong correlation between the used questions that can be grouped through factorial reduction. The KMO test value greater than 0,5 indicates a very good solution obtained through factorial analysis [57]. The p value associated with the Chi-Square test is 0 (< 0,01), indicating that with a probability of 99%, there are significant statistical links between the variables [57]. Since there are significant statistical connections between the variables, the factorial analysis of the main components was successfully applied [57].

Table 4 presents the matrix of the components after rotation for the multiple measures implemented to avoid the spread of the SARS-CoV-2 virus both within and outside the hospital. All (23) initially established factors were grouped into these 4 dimensions.

**Table 4:** Rotated component matrix of organizational measures.

No. crt.	Factors	Component				Cronbach’s Alpha
		C1	C2	C3	C4	
1	CIRCUL_SPACE	0,832				0,814
2	DELIMIT_SPACE	0,817				
3	SIGN_MAP	0,691				
4	SUPPLY_MADE	0,691				
5	AREAS3	0,569				
6	TRAINING_EMPLOYEE	0,564				
7	FORBID_ENTER	0,528				
8	SHIFT_STAFF	0,451				
9	RATIONAL_PPE		0,850			0,882
10	RULE_5HH		0,837			
11	PROTECT_AGP		0,826			
12	RESPIRATORY_PREV		0,754			
13	LIVING_SPACE			0,661		0,716
14	PSYCH_ASSIST			0,635		
15	FINANCIAL_COMPENS			0,624		
16	TRAINING_PREV			0,582		
17	CV_VACATION			0,554		
18	SUSPENTION_INTERACTION				0,786	0,804
19	QUARANTINE				0,747	
20	TEST_COVID19				0,659	
21	SELFM_TEMP				0,518	
22	INSTRUCT_SYMPT				0,486	
23	PREVENT_CONTACT_EXPOSURE				0,483	

Note: Extraction method: Principal component analysis. Rotation method: Varimax with Kaiser normalization. Rotation converged in 8 iterations. Source: authors’ contribution.

Based on the grouped factors, four main constructs were identified and named as follows:

**Component 1 — MANAGING THE DIVISION OF AREAS (contaminated and uncontaminated, COVID/ NON-COVID)** involves the separation of the main areas in the hospital (the area with COVID-19 patients and the care personnel, a potentially contaminated area and the uncontaminated space) and their proper marking. It involves restricting entry to third parties during infection outbreaks, organisation shifts for HCWs to reduce contamination risk, ensuring timely supply of necessary materials, and providing staff training on SARS-CoV-2 virus. Synthetically, it includes eight items: CIRCUL_SPACE, DELIMIT_SPACE, SIGN_MAP, SUPPY_MADE, AREAS3, TRAINING_EMPLOYEE, FORBID_ENTER, and SHIFT_STAFF.

**Component 2 — GENERAL PREVENTIVE MEASURES FOR HCWs WORKING IN CONTAMINATED AREAS** comprises wearing a mask, using disposable wipes for sneezing, washing hands after contact with nasal or oral secretions, etc.), the rule of the “5 important moments for hand hygiene” (before contacting the patient, before performing aseptic procedures, after exposure to body fluids, after touching the patient or after being in contact with objects near the patient). It also covers enhanced protective measures against airborne transmission during aerosol generating procedures (AGPs) for suspected or confirmed COVID-19 patients, and the rational use of personal protective equipment (PPE). Synthetically, it includes four items: RATIONAL_PPE, RULE_5HH, PROTECT_AGP, and RESPIRATORY_PREV.

**Component 3 — GENERAL STAFF MANAGEMENT MEASURES FOR HCWs WORKING IN CONTAMINATED AREAS** includes special living spaces arranged in the hospital for the medical staff directly exposed to COVID-19 patients, psychosocial assistance provided to staff in the unit during their quarantine/disease, financial compensation/ indemnities for the quarantine/ duration of the disease, advanced training/ education programs for infection prevention in HCWs, and compulsory or voluntary leave for non-COVID-related staff. Synthetically, it includes five items: LIVING-SPACE, PSYCH_ASSIST, FINANCIAL_COMPENS, TRAINING_PREV, CV_VACATION. Conceptually, all 5 items perfectly align with general managerial responsibilities and the exercise of managerial authority.

**Component 4 — MANAGING THE HEALTH OF THE HCWs WORKING IN CONTAMINATED AREAS,** comprising such measures as instructing staff to seek medical care if COVID-19 symptoms occur, applying preventive measures reducing contact and splash exposure, daily self-monitoring of temperature and respiratory symptoms, 14-day quarantine (if necessary), suspension of patient care interaction for a period of 14 days after unprotected exposure to a confirmed COVID-19 patient, and COVID-19. Testing. Synthetically, it includes six items: SUSPENTION_INTERACTION, QUARANTINE, TEST_COVID19, SELFM_TEMP, INSTRUCT_SYMPT, PREVENT_CONTACT_EXPOSURE.

To validate the items of the four components, internal consistency was tested using the Cronbrach’s Alpha coefficient. The items in all four components were homogeneous well-defined: C1 (α = 0,814 > 0,600); C2 (α = 0,882 > 0,600); C3 (α = 0,716 > 0,600); C4 (α = 0,804 > 0,600).

Each variable within the constructs was scored on a scale from 1 (minimum) to 5 (maximum) to assess the intensity of organizational measures implemented in the hospital. The mean scores for each construct were the calculated to test the research hypotheses. A test value of 4 was chosen, corresponding to “Most of the time”, representing 80% of adherence (100%). The results are presented in Table 5.

**Table 5:** Testing the research hypotheses (N = 312).

Constructs’ means	Min.	Max.	Mean	Std. Dev.	t	df	p	Mean Difference	95% Confidence Interval of the Difference	
									Lower	Upper
C1	2	5	4,79	0,422	33,303	311	0,000	0,795	0,748	0,842
C2	1,75	5	**4,87**	0,403	38,100	311	0,000	0,870	0,825	0,915
C3	1	5	3,40	1,066	−9,876	311	0,000	−0,596	−0,715	−0,477
C4	1,5	5	4,62	0,660	16,573	311	0,000	0,620	0,546	0,693

**Always (5)** = more than 95% of the time; **Most of the time (4)** = from 50% to 95% of the time; **Occasionally (3)** = from 20% to less than 50% of the time; **Rarely (2)** = less than 20% of the time; **Never (1)**; p < 0,001; Test value = 4; Source: authors’ contribution.

**Fig 1:**
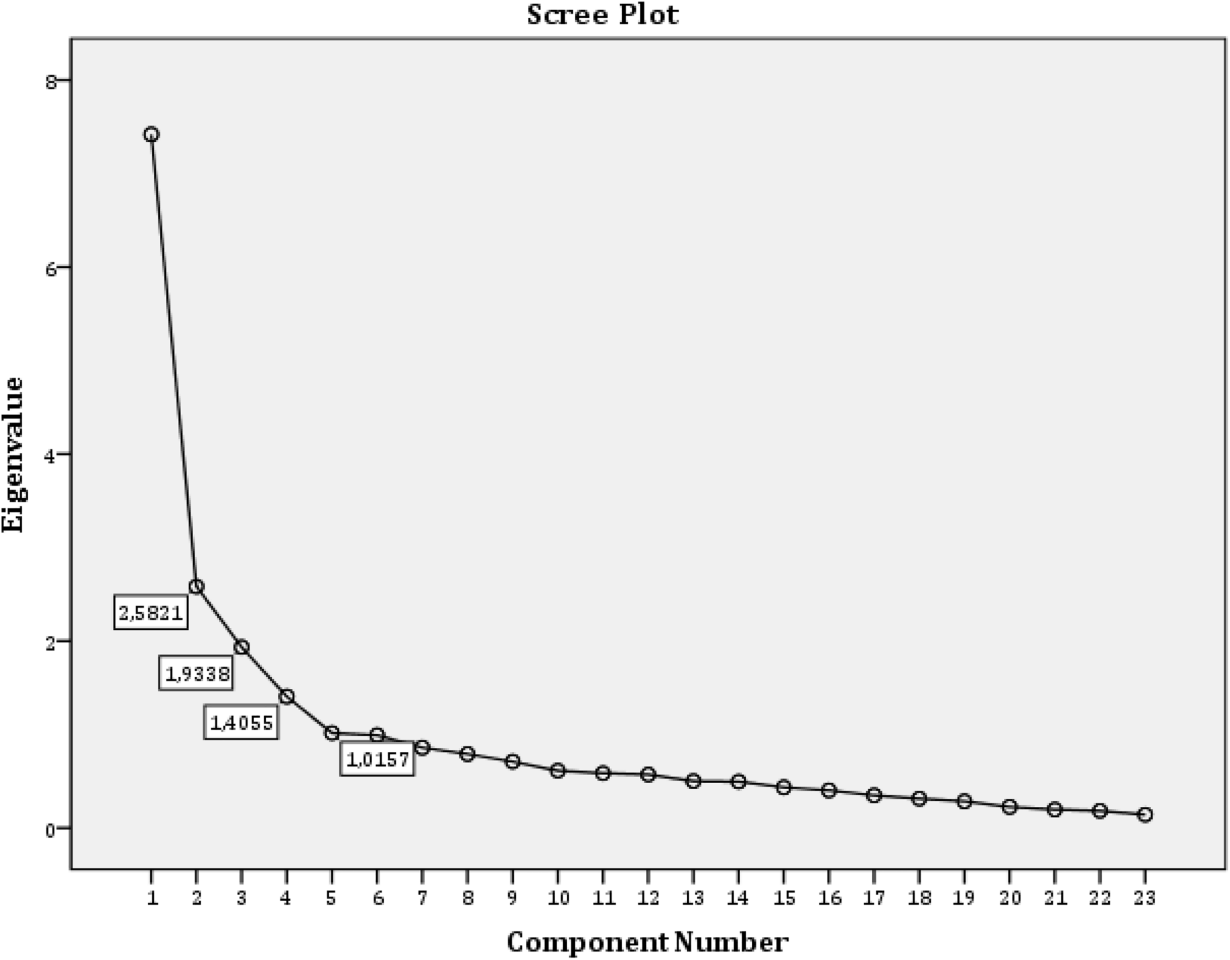
The Eigenvalues for the constructs of the research. (A) Caption credit authors’ contribution. The Eigenvalue (EV) explains the total variance of the constructs and shows positive statistical results if the values are greater than 1. For the first construct had an EV = 2,582 (>1), the second EV = 1,934 (>1), the third EV = 1,406 (>1), and the fourth EV = 1,016 (>1), which means that the constructs are reliable as defined.

These four components can be interpreted as layers of a security system designed to protect an “asset” from a threat, each layer contributing to progressively reducing the risk to a value close to zero. In this case, the “asset” to be protected is the health of the HCWs active in areas where the threat of catching a COVID-19 infection is significant. Fig 1 presents the four components and the correlations with the hospital’s four types of environments where HCW operate, thereby creating four layers of exposure risk management measures for the prevention and control of infections of healthcare workers treating COVID-19 patients.

**Fig 2:**
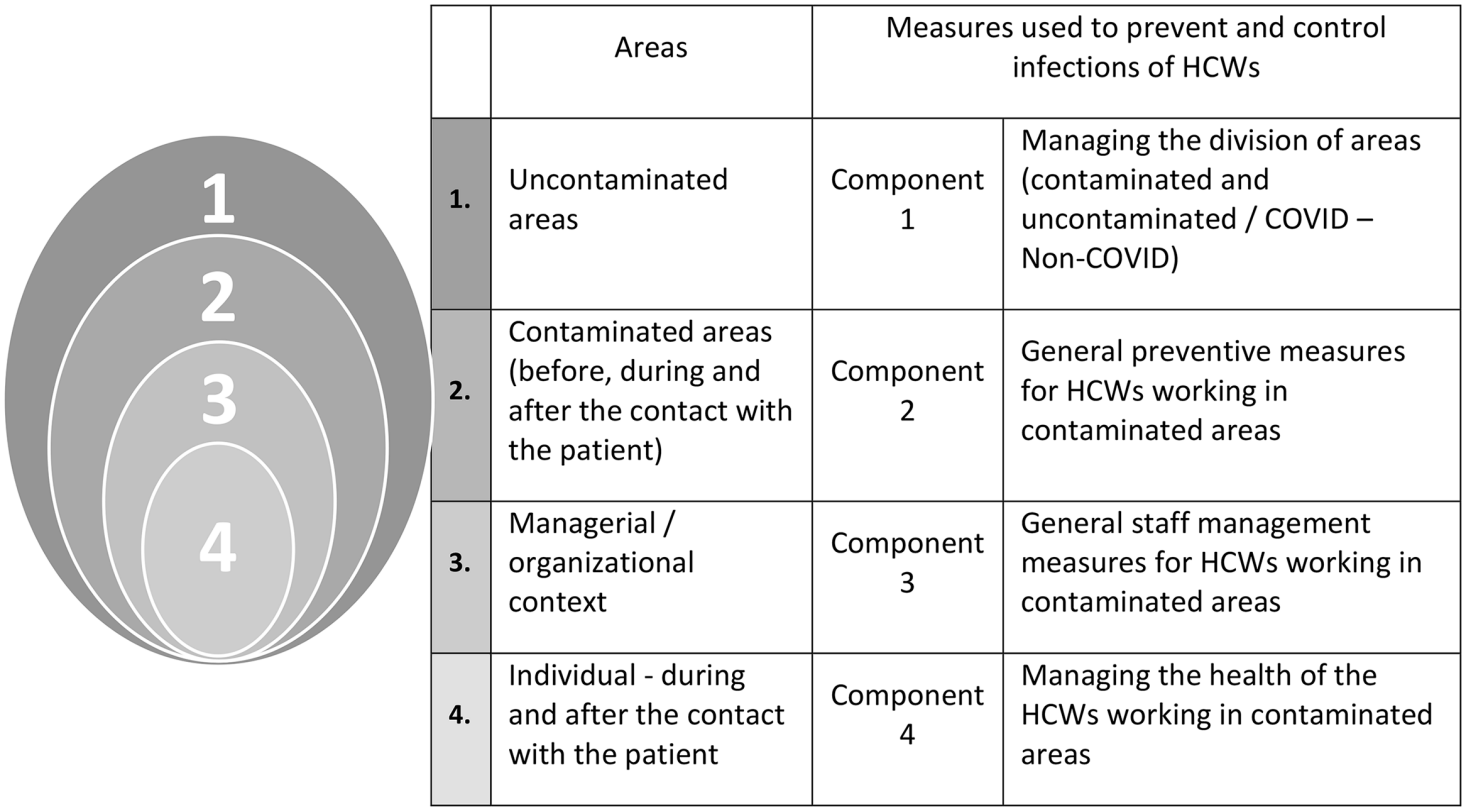
Four layers of exposure risk management measures for the prevention and control of infections of healthcare workers treating COVID-19 patients (based on the constructs defined with Principal Components Analysis). (A) Caption credit: authors’ contribution.

To conceptualise these layers, consider the healthcare worker’s journey through them during their duties: (1) starting from a low-risk uncontaminated area, (2) entering a contaminated area, (3) interacting directly with sick patients while performing medical procedures, (4) managing their own health before, during and after patient contact. Managerial interventions aimed at exposure risk management measures and infection prevention and control of infections are essential at each stage of these layers. All these managerial interventions should be designed and implemented in the hospital prior to the arrival at work of the HCWs (Fig 2).

*The first layer* of exposure risk management measures focuses on separating contaminated and uncontaminated areas through creation, implementation and enforcement of rules governing the movement of patients, HCWs and medical materials. The purpose of this layer is to protect the uncontaminated areas by containing the flow with potential contamination. It includes the establishment of grey zones or buffer zones for patients without a clear status.

*The second layer* comprises general preventive measures for HCWs operating in contaminated areas. Its purpose is to prevent any contamination of HCWs, when they are in contaminated areas, before, during and after contact with the COVID 19 patients. Measures related to wearing protective equipment are amongst the most important in this layer.

*The third layer* addresses organisational measures concerning staff attendance, including the provision of low-risk living spaces between shifts, alongside motivational, psychological and financial support, reduction of contamination risks through training and education. The goal of this layer is to mitigate health risks for HCWs by optimising work organisation.

*The fourth layer* focuses on individual-level risk management for healthcare workers in contaminated areas. This includes regular COVID-19 testing, general health monitoring and symptom reporting, potential quarantine or temporary suspension patient interaction.

Hypothesis testing was conducted using One-Sample Test (details in Table 5). All computed mean variables had significant p values: C1 (Mean = 4,79 out of 5; Std. dev. = 0,422; p = 0,000; t = 33,303); C2 (Mean = 4,87 out of 5; Std. dev. = 0,403; p = 0,000 < 0,001; t = 38,100); C3 (Mean = 3,40 out of 5; Std. dev. = 1,066; p = 0,000 < 0,001; t = −9,876); C4 (Mean = 4,62 out of 5; Std. dev. = 0,660; p = 0,000 < 0,001; t = 16,573). The t-test was performed with a test value of 4, corresponding to the response category „most of the time” (from 50% to 95% of the time), and the minimum mean of the constructs was 3,40 (C3).

Thus, the following hypotheses were validated:

*Hypothesis 1. Division of hospital areas into contaminated and uncontaminated zones, as well as into COVID and non-COVID sections, played an important role in managing the pandemic.*

*Hypothesis 3. Implementation of comprehensive support measures — including designated living spaces, psychological assistance, financial compensation, advanced training, and flexible leave options — were important in managing the pandemic.*

The highest mean among the four constructs was observed for C2 - *General preventive measures* – indicating that this was the most frequently applied set of measures. Measures such as consistent use of PPE, adherence to hand hygiene during patient contact, and enhanced protection during aerosol-generating procedures (AGPs) were implemented almost continuously (Mean = 4,87 out of 5; Std. dev. = 0,403), showing the highest mean. These findings confirm the second hypothesis:

*Hypothesis 2. General preventive measures applied in direct patient care were the most frequently implemented in the analysed hospital.*

Hypothesis 4 was validated by the results for construct C4 – „Managing the health of the HCWs working in contaminated areas” (Mean = 4,62 out of 5; Std. dev. = 0,660). The construct was considered crucial, as it addressed measures directly affecting HCWs with sustained patient contact:

*Hypothesis 4. The implementation of comprehensive health management measures for healthcare workers (HCWs) in contaminated areas, including suspension of patient care interactions after unprotected exposure, mandatory quarantine, regular COVID-19 testing and self-monitoring, and enhanced preventive practices, was crucial for managing the pandemic.*

## 5. Conclusions and discussions

The COVID-19 pandemic brought about changes in the management of healthcare institutions, primarily driven by legislative measures introduced by national governments or international health organization. These measures included mandatory COVID-19 testing, establishment of special triage zones, rescheduling of appointments for chronic patients, enforcing social distancing, mandatory mask use, and enhanced hand hygiene protocols.

The COVID-19 pandemic has been described as “tsunami” by De Donno et al. (2021), which triggered a series of managerial innovations aimed at maintaining permanent access to medical care [58]. Among the most significant developments was the widespread adoption of telemedicine and Remote Patient Monitoring (RPM), enabling patients to regularly self-report symptoms, oxygen saturation levels, and temperature [59]. Family medicine practitioners also adopted programs such as COVIDCare@Home, which provided support by tracking metrics including *adoption* (e.g., total number of visits per patient), *feasibility* (e.g., receiving an oximeter or thermometer), and *safety* (e.g., hospitalizations, mortality and emergency department visits) [60]. However, while the suspension of in-person interactions was critical for infection control, it also led to unintended negative consequences, including increased crime rates (such as theft, domestic violence, fraud) and long-term societal harm [61,62]. Conversely, increased digital interactions – particularly through social media platforms such as Instagram, YouTube, Facebook, Tik-Tok, Twitter – played a positive role in maintaining social connections. Notably, social media user numbers surged in 2021, with “*520 million new users joining worldwide within a 12month period up to July 2021, which equates to annualized growth of 13.1%, or an average 16.5 new users every single second*” [63].

Wearing masks remained one of the most widely recommended protective measures during the COVID-19 pandemic. Although vaccines became increasingly available, uptake varied, particularly among vulnerable populations with limited access, thereby contributing to continued virus transmission [64]. Gibson et al. (2023) observed discrepancies in the effectiveness of protective interventions, as outcomes were influenced by factors such as population characteristics, comorbidities, and demographic structure [65]. For example, „during *the Omicron wave, the effectiveness estimates for two doses of either Moderna or Pfizer mRNA vaccines ranged from 70% in a South African hospital and 36.6% across multiple testing facilities in Ontario, Canada*” [65,66]. The identification of specific symptoms and effective communication with family doctors played a crucial role in limiting the spread of the COVID-19 virus. To support this, digital symptom-type applications were developed, enabling real-time monitoring of disease progression. One such device, the symptom-type tracker, collected daily reported health data from both asymptomatic and symptomatic individuals, including symptoms, hospitalization, reverse-transcription PCR (RT-PCR) test results, demographic data, and pre-existing medical conditions. The application was launched in the United Kingdom on 24 March 2020 and in the United States on 29 March [67].

The logic underlying exposure risk assessment and management is comparable to that of a security system designed to protect an “asset” from a threat. In our case, the asset was the health of the HCWs at risk of infection while providing care to COVID-19 patients in the healthcare unit of the “Sf. Ioan cel Nou” County Emergency Hospital in Suceava, Romania. Based on the answers to our questionnaire, we sought to identify the main organisational measures implemented to prevent and control infections with COVID-19 patients in this hospital setting. Using Principal Components Analysis, four distinct components were identified, each grouping between 4–8 exposure risk management measures.

These four components may be interpreted analogously to a security system designed to protect an “asset” from a threat, using several layers to gradually reduce the associated risk to a value close to zero. In this case, the “asset” to be protected is the health of the HCWs operating in areas where the threat of catching a COVID-19 infection is significant.

We were surprised by the perfect parallelism found between the practical sequence of environments traversed by HCWs when treating COVID-19 patients and the four components resulting from the PCA. The HCW transitions from a low risk, uncontaminated area to a contaminated area, comes into a direct contact with COVID-19 patients while performing medical procedures, and manages their own medical status during and after such contact. The four constructs derived from the PCA follow exactly the same logic. This parallelism enabled a deeper understanding of the previously unseen and unarticulated four-layer protection system set up to prevent and control the infections among HCWs working in the hospital.

The first layer of exposure risk management measures is designed to contain the COVID-19 virus in separated areas by establishing, implementing and enforcing, when necessary, the rules related to contaminated areas, grey zones, and uncontaminated areas, as well as the flows of patients, HCWs, and medical materials.

In contaminated areas, the reduction of health risks for HCWs relies on general preventive measures, such as the wear of protective equipment. This is the second layer of measures aimed at preventing any contamination of HCWs before, during and after contact with COVID 19 patient.

The third layer is primarily managerial in nature and is aimed at reducing health risks for or the HCWs by optimising work organisation. Preventive measures in this layer include the regulation of staff attendance at work, provision of low contamination risk living spaces between shifts, motivational, psychological and financial support, as well as the reduction of contamination risks through training and education.

The fourth layer encompasses health issues at individual level. It includes measures applied personally by HCWs working in contaminated areas, such as periodic COVID-19 testing, general health monitoring and symptom reporting, temporary suspension of interaction with patients, and quarantine.

Hypothesis testing showed that all measures applied in the hospital were important in managing the pandemic, indicating that a functional exposure risk management system must ensure effective risk reduction in all 4 layers. Yet, the confirmation of the second hypothesis, namely, that the most frequently applied measures in the analysed hospital were the general preventive ones – suggests that both HCWs and managers might tend to put an emphasis on this type of preventive measures, limited only to the second layer. From a risk management perspective, we can speculate that this can potentially lead to an uneven effort, with more managerial attention and resources being given to the second layer, compared to the other three layers.

For this reason, we believe that our study contributed to illustrating the existence of four distinct layers of exposure risk management measures, which have the potential to assist hospital managers in designing and managing well- balanced similar systems when confronted with any type of infectious contagion. A clear understanding of each layer in the overall reduction of the HCWs health risks constitutes a foundation for a well-balanced healthcare management system.

To assess the originality of our exposure risk management model, we reviewed the guidelines published by World Health Organization [39,40,51,68], the US Centres for Disease Control and Prevention (CDC) [69], and the European Centre for Disease Prevention and Control (ECDC) [70]. All three provide comprehensive lists of guidelines, recommendations or rules to be applied, most of which focus on hygiene practices for individuals, spaces and equipment. However, none of these adopt a layered perspective. A managerial or organizational dimension partially aligned with our Component 3 can be found only in the ECDC guidelines for Covid-19 in the subchapter “Health monitoring and management of exposed staff”, and in the CDC guidelines concerning “Infection Control in Healthcare Personnel: Infrastructure and Routine Practices for Occupational Infection Prevention and Control Services (2019)” [69,70]. Therefore, the novelty of our proposed model lies in two conceptual contributions: (1) the introduction of layered risk reduction approach, similar to a security system designed to reduce an external threat; and (2) the integration of managerial perspective into risk reduction, explicitly incorporating organizational activities within the protection layers. Other models predominantly adopt a health or medical perspective, often overlooking the managerial and organizational dimensions.

Paying attention to wearing the protective equipment is highly important but not enough. It is rational to design a system that starts with the first potential source of contamination – the direct contact with the sick patient – but from a managerial point of view, the contamination is not a punctual but a systemic problem, requiring a process management perspective. Hence, correct flows for keeping the contaminated and uncontaminated areas separated, managerial organisation of work in a manner that reduces contamination risks, and managing the health of the HCWs working in contaminated areas through COVID-19 testing, general health monitoring, symptom reporting, temporary suspension, or even quarantine are equally important for the success of the system.

The analysis of the links between the component elements of a managerial process adapted to the COVID-19 situation, from the perspective of managerial efficiency and effectiveness, comprises all the specific elements identified through the analysis of the main components included within the defined layers: the uncontaminated area (spaces for patient care, protection zones, and areas with restricted access), the contaminated area (includes general prevention measures, rules, employee training), managerial organizational context (financial compensation, testing, etc.); and the individual dimension during and after contact with the patient (quarantine, suspension of interaction).

This study presents several limitations that should be acknowledged. First, the use of a self-administered, retrospective questionnaire introduces the risk of response bias, particularly social desirability bias. HCWs may have overstated their adherence to protective measures or understated negative experiences to align with institutional expectations during the COVID-19 crisis. Second, the questionnaire was adapted specifically to the context of “Sf. Ioan cel Nou” County Emergency Hospital in Suceava, Romania. While this ensured local relevance, it limits the generalizability of the findings to other hospitals or healthcare systems with different conditions, resources, or management practices. Third, the exclusive use of online survey may have resulted in sampling bias, as HCWs with limited access to digital tools or insufficient digital literacy may have been unintentionally excluded, thereby affecting the representativeness of the sample. Finally, although the proposed “four-layer” conceptual model has clear practical significance, it has not undergone formal statistical validation.

## Supporting information

S1 File Database. (XLS)

S2 File Output. (XLS)
