# Correction: Layers of exposure risk management measures for the prevention and control of infections of healthcare workers treating COVID-19 patients

**DOI:** 10.1371/journal.pone.0340506

**Published:** 2026-01-06

**Authors:** Neştian Ștefan Andrei, Turnea Elena-Sabina, Tiţă Silviu-Mihail, Vodă Ana-Iolanda, Poroch Vladimir

The author initials appear incorrectly in the citation. The correct citation is: Neştian AS, Turnea ES, Tiţă SM, Vodă AI, Poroch V (2025) Layers of exposure risk management measures for the prevention and control of infections of healthcare workers treating COVID-19 patients. PLoS One 20(9): e0332804. https://doi.org/10.1371/journal.pone.0332804.

Additionally, the ORCID iDs are missing for the first, third, fourth, and fifth author. Please see the authors’ respective ORCID iDs here:

- Author Neştian Ștefan Andrei’s ORCID iD is: 0000-0002-3499-3245 (https://orcid.org/0000-0002-3499-3245).
- Author Tiţă Silviu-Mihail’s ORCID iD is: 0000-0003-2817-9199 (https://orcid.org/0000-0003-2817-9199).
- Author Vodă Ana-Iolanda’s ORCID iD is 0000-0003-2306-0172 (https://orcid.org/0000-0003-2306-0172).
- Author Poroch Vladimir’s ORCID iD is 0000-0002-2503-5395 (https://orcid.org/0000-0002-2503-5395).
